# *On* Ischuria, *the Introduction of the* Catheter, *and Most Expeditious Method of Making* Leeches *Bite*

**Published:** 1805-11-01

**Authors:** 

**Affiliations:** Surgeon of His Majesty's Ship Pegasus


### *On* Ischuria, *the Introduction of the* Catheter, *and most expeditious Method of making* Leeches *Bite*; *by* Dr. Cuming, m. d. *Surgeon of His Majesty's Ship Pegasus.*

CONCEIVING that the enclosed case may be deemed interesting to many of your readers, and beneficial to some unhappy sufferer, labouring under the same complaint, though there is nothing very nouvelle in the treatment, without the mode adopted for the introduction of the catheter may be esteemed such. Be this as it may, I have great pleasure in recommending this method of introducing the instrument, from a thorough conviction in my own mind, that the operation will in general succeed, and be greatly facilitated by it.

Having mentioned the application of leeches to the perinæum, I shall avail myself of this opportunity of pointing out an easy and expeditious way of making them bite, an operation fraught, not unfrequently, with great tædium and perplexity, and which often exhausts the patience of the operator. For this knowledge, I am indebted to a man in Romsey, at whose dexterity I was at first surprised. He is a dealer in leeches, and generally applies them himself, receiving the market price for them, and the leeches, after they have performed their office, for his trouble.

He ties up the end of one of his fingers with a piece of thread or tape, and plunges the point of a needle into it, and the blood which is drawn, he applies to the part which it is intended the leeches should bite. But before the application of the blood, the part is well washed, and freed from the scent or grease of any medicament which may have been previously applied. If it be necessary to wash it with soap, it will be adviseable to rewash with milk afterward. Any number may be almost instantaneously fixed in this way, provided the tail of the animal is not taken for the head; and I must here remark, that there is no difficulty in discovering the one from the other; the head is always on the stretch when handled, is quite pointed, and of a conical shape, so that, as soon as the finger and thumb are applied to the body of the animal, the head will ever be discovered from its appearing smaller and more pointed than the tail; and when it tastes the blood, it in general fixes on the spot immediately.

I have known them applied in this manner as fast as the operator could pick them up from the vessel which contained them. One of the greatest advantages resulting from this knowledge, consists in being enabled to draw blood from the very point where it is most required. I have been frequently foiled in my attempts to fix them where I wished, and have sacrificed much time and patience in the attempt, previously to my having been favoured with this fortuitous sapience. Those who know how to appreciate local bleeding in topical inflammation, and conjestion of blood, in apoplexy for instance, will not think the time employed in reading this paper mispent; and if I mistake not, the learned Editors of this work will readily find a place for any communication which may either directly or indirectly add any thing to the stock of medical knowledge, or benefit the ars medendi.

Tempus fugit et fugit celeriter! Therefore let such of the profession who have treasured up knowledge, that may be useful to their fellow labourers, and beneficial to the community, impart it freely, and not, miser-like, shut it up in the rusty coffers of their narrow-minded souls.

ON the 14th of Nov. 1803, Mr. C—— made application to me for relief from a suppression of urine, stating, that for many hours he had not been able to void more than a few drops at a time, and that his inclination was constant, and the pain and irritation extremely severe; he was of a plethoric, sanguine temperament, in his 22d year, muscular, and well formed. I gave him pulv. jalap. gr. xxv. sal. nitr. ℈j. statim sumendus. Five or six hours elapsed without any evacuation; half the quantity of the same medicine was repeated, and in the evening a copious discharge took place, without affording any relief, the bladder was greatly distended, and the parietes of the abdomen above the os pubis were tense and prominent. I advised himto submit to the the introduction of the catheter, which he readily consented to, but was foiled in my attempt, after trying every possible method that was most likely to render my endeavours efficient; the catheter entered with great ease, apparently as far as the membranous part of the urethra, or to the prostate gland; and by using a steady, firm, and cautious force, I felt the sensation of something giving way to the point of the instrument, impressing me with the idea that bands were formed across the passage; from the exudation of coagulable lymph, it is equally probable that the passage was nearly or totally obliterated by surrounding inflammation: the patient possessed more than ordinary fortitude, and begged me to persevere. I found the instrument advance suddenly for the space of half an inch, which induced me to hope that the stricture had been overcome, but to my great regret, on withdrawing the stillette, nothing but a few drops of blood followed.

I now deemed it prudent to desist; on removing the catheter, about an ounce of urine followed with very hard straining. I immediately gave directions for a warm bath to be prepared, and in the interim he took tinct. opii, gtt. 50, and before he was put into the bath I bled him from a large orifice to the amount of ℔j. when he was immediately put into a large oval tub, and my reason for preferring this form of the vessel will appear in the sequel. He was kept in the bath in an horizontal posture for forty-one minutes, when he became exceedingly faint, and begged to be put to bed; his face was completely covered with perspiration, and there is every reason to think that the perspiration on the rest of his body had been very considerable, as the pain was considerably abated. I suppose he evacuated a small quantity of urine in the bath, for I advised him to make efforts for that purpose, though on examination, after he was put to bed, the same degree of tenseness and prominence was observed above the os pubis. I left him, with directions to drink as little as possible of barley water, or any other diluent, and desired him to moisten his mouth with a slice of lemon and loaf sugar; he had another anodyne draught to take during the night. I did not think it prudent to risque a repetition of the introduction of the catheter, for fear of inducing great inflammation, especially as the current of the fluids had been directed to the surface, on which account I consoled myself with the idea that the secretion from the kidnies would be trivial; and from the plans I intended putting in execution tion in the morning, after the body had been so perfectly depleted, I augured favourably of the result of the case.

15th. I found his pulse full and frequent, his tongue nearly covered with a white fur; he had a restless night, and voided a few drops of urine at intervals; the distention of the bladder appeared much the same, and the pain was very distressing. I had the patient again put into the warm bath, and kept him there until he was so much relaxed, that deliquium seemed to be fast approaching; this I deemed a favourable opportunity for the introduction of the catheter, and therefore took advantage of it whilst he remained in the large *oval tub*, for herein the advantage of this sort of vessel must appear obvious; the tub was deep, and from the quantity of water it contained for the purpose of completely covering the patient, it was necessary to place a small stool under the breech of the patient; and whilst the upper part of his body was supported by resting his arms on the edge of the tub, the extremities were drawn up, for the sake of relaxing the body, and thereby facilitating the introduction of the cather; and notwithstanding every thing appeared to favour my exertions, yet I met with considerable resistance, and sufficient to stagger my fortitude, but by a cool and steady perseverance I had the unspeakable felicity of seeing my endeavours crowned with succcess, and, under the blessing of Providence, I snatched from the jaws of death a lieutenant of the navy, whose valuable life may benefit his country, and bind his brow with never-fading laurels. I drew off nearly a large chamber-pot full of high coloured urine, had him immediately wiped dry, and put into a warm bed; the catheter was secured by passing a tape round him, and it remained several hours in the passage. By way of a refrigerant I gave him pulv. sal. nitr. ℈j. 4 de die, allowed him a poached egg, and such liquids as were diluting and aperient, with injunctions to be sparing in their use. When I visited him in the evening, very little urine had been discharged; he complained of little pain, and was as well in every respect as circumstances would admit of; the sensations he felt in the urethra, he said, were strange, and he experienced some pain and numbness in the course of the sciatic or crural nerve, which took place, and continued from the first time I attempted to draw his water off. Though little urine had been discharged during the day, I was not alarmed, for, from the relaxation observable above the pubes, I imagined little had been secreted. The anodyne was repeated at bed time.

 16th

16th. He enjoyed a pretty good night, and voided his urine in a small stream; the same treatment was observed with respect to medicine and regimen: in the evening, a messenger was dispatched after me, with the unpleasant tidings, that the patient's water came off very slowly, and with great difficulty; on visiting him, he complained of a sense of heat and fullness about the neck of the bladder; dreading a repetition of the inflammation, nine or ten leeches were immediately applied to the *perinæum*, which discharged a considerable quantity of blood, and relieved him so much, that his urine, in the space of a few hours, flowed in a full stream. The relief obtained in this instance, was so evident, that I think it a duty incumbent on me to recommend their application to this part in all cases of ischuria, arising from inflammation. This, if I may be permitted to expresss myself, is a piece of utility which neither teachers of medicine nor authors have inculcated in this disease.

17th. He passed a comfortable night, and in a few days was restored to perfect health.

This disease is universally allowed to be very dangerous; and from the practice I have had in it, I am inclined to suppose, that many cases have terminated fatally from want of perseverance and fortitude on the part of the surgeon. The introduction of the catheter is certainly a painful operation, yet, in my opinion, in all cases of imminent danger, the surgeon should be deaf to the intreaties of the patient, when he desires him to desist from pursuing those means, which alone can insure success; for that principle which arises from a conscientious discharge of duty, should never yield to motives of an inferior nature, such as may be denominated tenderness, or false delicacy; many opportunities of showing his conciliatory kindness and attention will occur, without risking the life of his patient by cowardly concessions.

*September* 15, 1805.

